# A Chinese female Morvan patient with LGI1 and CASPR2 antibodies: a case report

**DOI:** 10.1186/s12883-016-0555-x

**Published:** 2016-03-16

**Authors:** Li Zhang, Qiang Lu, Hong-Zhi Guan, Jun-Hua Mei, Hai-Tao Ren, Ming-Sheng Liu, Bin Peng, Li-Ying Cui

**Affiliations:** Department of Neurology, Peking Union Medical College Hospital, Chinese Academy of Medical Sciences and Peking Union Medical College, Shuaifuyuan 1, Dong Cheng District, Beijing, 100730 China; Department of Neurology, Wuhan First Hospital, Hospital of Integrated Traditional and Western Medicine, Tongji Medical College of Huazhong University of Science and Technology, Wuhan, 430022 Hubei Province China; Neuroscience Center, Chinese Academy of Medical Sciences and Peking Union Medical College, Shuaifuyuan 1, Dong Cheng District, Beijing, 100730 China

**Keywords:** Morvan syndrome, Leucine-rich glioma inactivated protein 1 antibodies, Contactin associated protein-like 2 antibodies, Limbic encephalitis, Voltage-gated potassium channels

## Abstract

**Background:**

Morvan syndrome is a rare disorder characterized by the combination of peripheral nerve hyperexcitability, encephalopathy and dysautonomia with marked insomnia. It was reported to have association to antibodies to voltage-gated potassium channels including contactin associated protein-like 2 antibodies (CASPR2-Ab) and leucine-rich glioma inactivated protein 1 antibodies (LGI1-Ab). LGI1-Ab was reported to associate with seizures, amnesia, confusion, hyponatraemia and a good prognosis, while CASPR2-Ab with peripheral presentations, probable risk for tumor and a poor prognosis. The vast majority of Morvan syndrome patients were male, with normal magnetic resonance imaging of the brain.

**Case presentation:**

We report a female case presenting with a combination of bilateral leg pain, widespread myokymia, memory disturbance, seizure, hyperhidrosis and insomnia. She had antibodies targeting CASPR2 and LGI1, tested by the indirect immunofluorescence test, which demonstrated the diagnosis of typical Morvan syndrome as well as classical limbic encephalitis. Cranial MRI revealed bilateral hyper-intensity of the medial temporal lobe, insular lobe and basal ganglia on T2/FLAIR and DWI sequence. As the treatment carried on, her serum LGI1-Ab disappeared and her memory loss, seizure and confusion quickly relieved. But her peripheral presentations did not relieve until serum CASPR2-Ab turned negative. Intravenous immunoglobulin treatment showed limited efficacy while she achieved almost complete remission with corticosteroids therapy.

**Conclusions:**

This case provides a rare female resource of Morvan syndrome, which is the first patient with both CASPR2-Ab and LGI1-Ab positive Morvan syndrome in China and one of the few female patients with Morvan syndrome reported so far. Through the detailed analysis of her clinical course, the diverse and overlapping clinical phenotype of CASPR2-Ab and LGI1-Ab in patients with Morvan syndrome was obvious and interesting.

## Background

Limbic encephalitis (LE) is defined as the subacute development of seizures, short-term memory loss, confusion and psychiatric symptoms suggesting the involvement of the limbic system [1]. Peripheral nerve hyperexcitability (PNH) is used to describe acquired neuromyotonia (NMT) or partial manifestations of this disorder including cramps, muscle twitching (fasciculations or myokymia) and muscle stiffness [2, 3]. And Morvan syndrome is a rare disorder characterized by the combination of PNH or NMT, encephalopathy and dysautonomia with marked insomnia [4]. The vast majority of Morvan syndrome patients were male, with normal magnetic resonance imaging (MRI) of the brain [4]. Plenty of studies have demonstrated the association between Morvan syndrome and antibodies to voltage-gated potassium channels (VGKC-Ab) including contactin associated protein-like 2 antibodies (CASPR2-Ab), leucine-rich glioma inactivated protein 1 antibodies (LGI1-Ab) and other antibodies [2, 4–8]. LGI1-Ab was reported to associate with seizures, amnesia, confusion, hyponatraemia and a good prognosis, while CASPR2-Ab with peripheral presentations, probable risk for tumor and a poor prognosis [2, 4, 9].

Here we report on a both CASPR2-Ab and LGI1-Ab positive female patient who presented with typical Morvan syndrome as well as classical LE, which is the first patient with double antibodies positive Morvan syndrome in China and one of the few female patients with Morvan syndrome reported so far. She had an abnormal cranial MRI, and and achieved almost complete remission with the treatment of steroids and IVIG. Through the detailed analysis of her clinical course, we aim to emphasis the diverse and overlapping clinical phenotype of LGI1-Ab and CASPR2-Ab in patients with Morvan syndrome.

## Case presentation

**Fig. 1:**
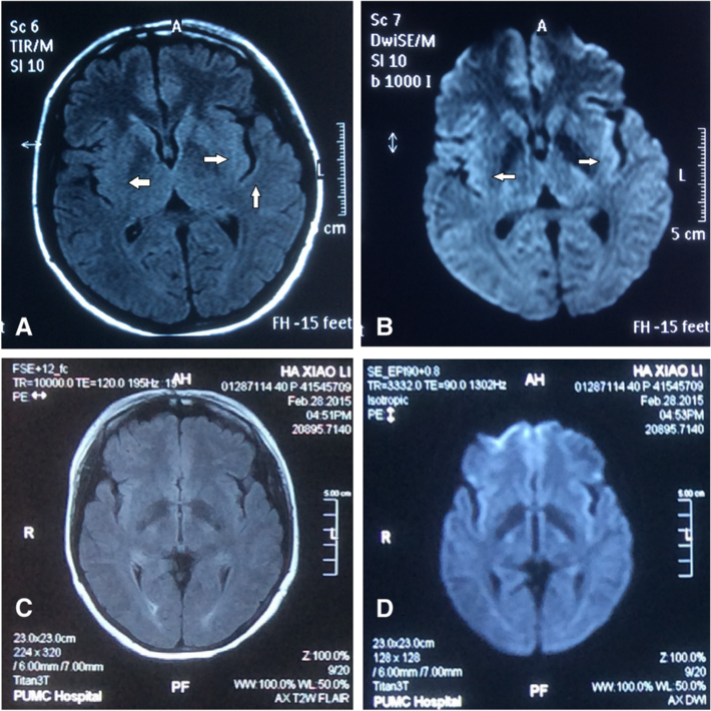
Cranial MRI of our patient. Diffusion-weighted magnetic resonance imaging (DWI) (**a**) and the corresponding plane in fluid-attenuated inversion recovery (FLAIR) (**b**) showed bilateral hyper-intensity of the medial temporal lobe, insular lobe and basal ganglia (arrows). Repeated MRI were normal in February 28, 2015. (**c**, **d**)

A 40-year-old Chinese woman presented with a 2-month history of bilateral leg pain, widespread myokymia, memory disturbance, seizure, hyperhidrosis and insomnia. Two months prior to the admission, she developed repeating pain in bilateral proximal end of lower limbs, accompanied with widespread myokymia, shaking of the toes, insomnia and hyperhidrosis. One week later, she developed seizures for three times a day, consisting of eyes on the turn, froth at the mouth, unresponsiveness and convulsion of bilateral upper limbs. Levetiracetam was started by the referring doctor, which relieved her seizure soon. From then on, family members noted her recent memory loss, confusion and apathy. Cranial MRI (Fig. 1) revealed bilateral hyper-intensity of the medial temporal lobe, insular lobe and basal ganglia on T2/FLAIR and DWI sequence. Electroencephalography (EEG) showed a few non-specific slow waves in background and occasional abnormal sharp waves on occipital lead during wakefulness.

**Fig. 2:**
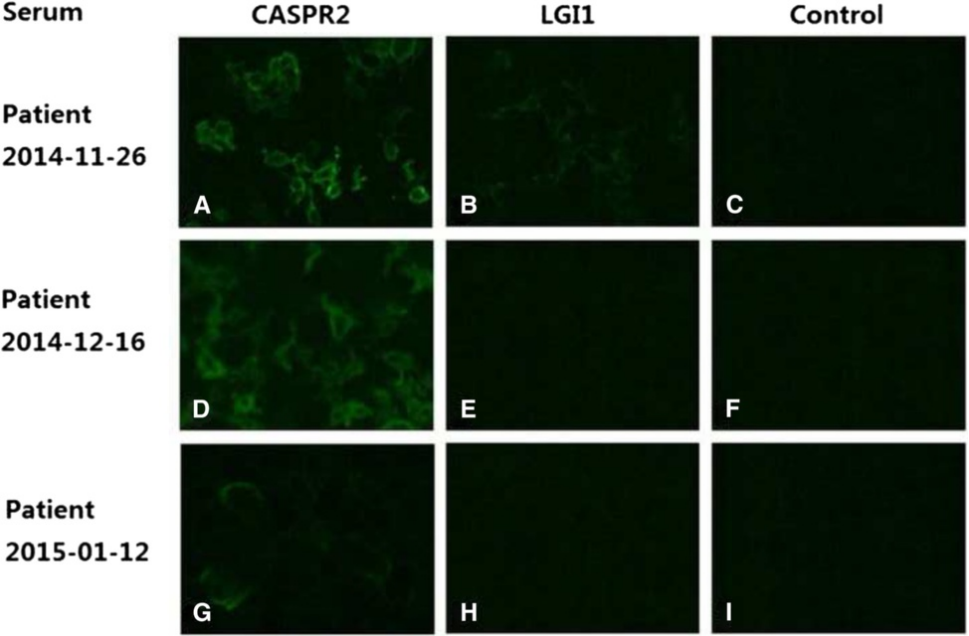
The immunoreactivity of patient’s serum to CASPR2 and LGI1 proteins. EU90 cells were transfected with cDNAs encoding CASPR2, LGI1 and other four neuropil proteins associated with autoimmune encephalitis (EUROIMMUN, FA 112d-1005-1, Germany), incubated with this patient’s serum and detected by IIFT. This patient’s serum collected at different time showed different immunoreactivity to CASPR2 (**a**, **d**, **g**) and LGI1 (**b**, **e**, **h**), and areas with no specific fluorescene to other proteins on the same slide tested at the same time would be regard as control (**c**, **f**, **i**). The patient’s serum collected on November 26, 2014 showed strong binding to the surface of cells expressing CASPR2 proteins (**a**) (50x) and moderate binding to the ones expressing LGI1 proteins (**b**)(50x). The patient’s serum collected on December 26, 2014 showed moderate binding to the surface of cells expressing CASPR2 proteins (**d**) (50x) and no binding to the ones expressing LGI1 proteins (**e**) (50x). The patient’s serum collected on January 12, 2015 showed weak binding to the surface of cells expressing CASPR2 proteins (**g**) (100x) and no binding to the ones expressing LGI1 proteins (**h**) (100x)

Serum test in a cell based assay showed serum VGKC-complex proteins (EUROIMMUN, Germany) including CASPR2-Ab strongly positive (+++) and LGI1-Ab positive (+), while cerebrospinal fluid (CSF) LGI1-Ab was weakly positive (±), examined by the indirect immunofluorescence test (IIFT) (Fig. 2). CSF for cells, glucose, chloride and culture were normal, while CSF protein was mildly elevated at 0.5 g/L. Blood natrium was normal. Thyroid function and anti-thyroid antibodies including Anti-TG and Anti-TPO was negative. Tumor markers (CEA, AFP, CA125, CA19-9, CA15-3, SCCAg, NSE, Cyfra211, TPS) and paraneoplastic neuronal antibodies (Hu, Ri, Yo) were all negative, and body CT scanning showed no malignancy. Her mini-mental state examination (MMSE) was 21/30. Neurologic examination revealed only myokymia in the limbs, shaking of the toes, and hyperhidrosis. She has no remarkable past history, personal history or family history.

**Fig. 3:**
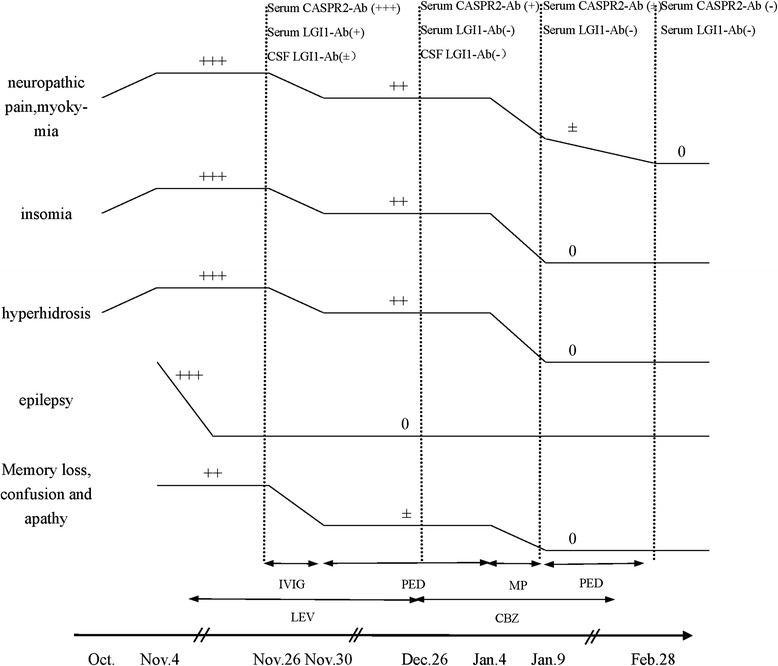
Timing of clinical course, antibodies test and treatment. 0, No symptom; +, mild; ++, moderate; +++, severe; IVIG, intravenous immunogloblin; PED, prednisone; MP, methylprednisolone; LEV, Levetiracetam; CBZ, carbamazepine

VGKC-Ab related limbic encephalitis and Morvan’s syndrome were presumed diagnosis. She was treated with intravenous immunoglobulin (IVIG) at a dose of 0.4 g/kg/day for 5 days and continued oral prednisolone 20 mg/day treatment for a month. Carbamazepine 0.1 g tid was administered instead of levetiracetam to prevent epilepsy recurrence. Her memory loss and confusion responded quite well to the immunotherapy, while bilateral leg pain, myokymia, shaking of the toes, insomnia as well as hyperhidrosis mildly relieved but not disappeared. Repeated CSF routine, cells, glucose, chloride, protein, CASPR2-Ab and LGI1-Ab were within the normal range. Serum CASPR2-Ab was still positive (+) while LGIA-Ab turned negative. Electromyography (EMG) revealed abnormal F wave in bilateral lower limb, indicating abnormal peripheral nerve excitability. Nerve conduction velocity showed normal motor and sensory nerve responses, and somatosensory evoked potential (SEP) of lower limbs were normal. Methylprednisolone pulse therapy (1 g for 3 days and 500 mg for 3 days) was commenced, after which all symptoms above got significant remission. Repeated MMSE was 27/30. Repeated serum CASPR2-Ab turned weakly positive (±). Prednisolone 60 mg daily oral treatment was administered subsequently with slow tapering (Fig. 3). She did a good job in following the treatment plan and showed no specific adverse reaction. In a 50 days’ follow-up, after stopping all the drugs for almost 1 week, her repeated MRI (Fig. 1) were normal and all of her symptoms experienced complete remission.

## Methods

Serum from the patient was qualitatively tested for neuropil antibodies associated with autoimmune encephalitis including antibodies to glutamate receptors type NMDA, type AMPA1 and type AMPA2, LGI1, CASPR2 and GABARB1/B2using the indirect immunofluorescence test (IIFT) (EUROIMMUN, FA 112d-1005-1, Germany). Cell-based assays (CBAs) for those antibodies were performed using EU90 cells (EUROIMMUN) transfected with cDNAs encoding the relevant proteins. Combinations of substrates were incubated with patient serum (1:10 dilution) or undiluted CSF sample. In a second step, the attached antibodies were stained with fluorescein-labelled anti-human antibodies (EUROIMMUN) and made visible with a fluorescence microscope. Each slide was devided into six areas detected for the above-mentioned six antibodies and those areas with no specific fluorescene would be regard as the control of positive area with specific fluorescence. Fluorescence intensity level was used to describe the intensity of the specific fluorescence as a numeric value, reaching from “0” or “−” (no specific fluorescence) to “5” or “+++++” (extremely strong specific fluorescence). The deviation in the fluorescence intensity of the IIFT amounted to no more than ± 1 fluorescence intensity level for all samples.

## Discussion

There are very few female Morvan syndrome cases with both CASPR2-Ab and LGI1-Ab positive [1, 7, 10]. Loukaides et al. [8] have reported a 67-year-old man with autoantibodies targeting LGI1, CASPR2 and Contactin-2/Tag-1 presenting with Morvan syndrome. Klein et al. [7] and Lai et al. [9] have reported 9 patients whose CASPR2-Ab and LGI1-Ab were both positive, but none of them were diagnosed with Morvan syndrome. Although 29 Morvan syndrome cases have been collected by Irani et al. [4], of which only two patients were female.

Our patient’s symptoms can be devided into following aspects. Neuropathic pain, widespread myokymia and shaking of the toes were manifestations of PNH [2, 3]. And epilepsy, recent memory loss, confusion and apathy were classified as symptoms of limbic encephalitis (LE) [1, 4, 9]. With hyperhidrosis as dysautonomia and insomnia, the patient was diagnosised with a typical Morvan syndrome [4]. Compared with LE, Morvan syndrome showed significant less amnesia, confusion or seizures as the neuropsychiatric manifestations, but more hallucinations and agitation. Whereas the presence of neuromyotonia, dysautomia (hyperhidrosis, cardiovascular) and neuropathic pain would help to distinguish Morvan syndrome from classical LE [4]. In addition, most Morvan syndrome patients were reported with normal cranial MRI findings [4, 8]. For this patient, we would like to attribute the encephalopathic clinical features as well as abnormal intensity on cranial MRI to the common phenomenon of LE. Thus this patient should be diagnosed with classical LE as well as Morvan syndrome.

There was a detailed change of the clinical manifestation and titers of antibodies along with the treatment. In the beginning, her serum CASPR2-Ab was strongly positive (+++) and LGI1-Ab was positive (+), while CSF LGI1-Ab was weakly positive (±). After the treatment of IVIG, both her serum and CSF LGI1-Ab turned negative, and in the meanwhile her memory loss, confusion and apathy resolved to some extent. At the same time, her serum CASPR2-Ab kept positive, from which we could conclude that LGI1-Ab was the primarily relevant antibody for these encephalopathic symptoms. In this case we can’t arbitrarily contribute the seizure to LGI1-Ab for the timely treatment with antiepileptic drugs since the beginning of seizure. After the methylprednisolone pulse therapy, her symptoms of PNH, insomnia and hyperhidrosis almost disappeared while serum CASPR2-Ab turned weakly positive. Besides, titer of CASPR2-Ab was always higher than that of LGI1-Ab, which indicated the association of CASPR2-Ab with PNH and Morvan syndrome. This agrees with the different clinical features of CASPR2-Ab and LGI1-Ab that CASPR2-Ab was in association with PNH and Morvan syndrome while LGI1-Ab with memory loss, cognitive impairment and seizures [2, 6, 7, 9].

The combination of LGI1-Ab and CASPR2-Ab attracted our attention. Within the 29 Morvan cases reported by Irani et al. [4], there were 15 patients with both positive LGI1-Ab and CASPR2-Ab, among which there were three patients with additional contactin-2 antibodies. Different antibodies may contribute to the distinct phenotype of Morvan syndrome. Our case also corresponded with the condition that Morvan syndrome is usually associated with high-titer CASPR2-Ab and often accompanied by lower-titer LGI1-Ab [4]. From her treatment and clinical course, the diverse and overlapping effects of LGI1-Ab and CASPR2-Ab in Morvan syndrome were apparent.

Irani et al. [4] have described 29 Morvan syndrome cases, of which 27 patients were all male. And all five Morvan syndrome patients reported by Sarosh et al. [6] were male. Seven of eight patients with positive CASPR2-Ab collected by Lancaster et al. [2] with or without Morvan syndrome were male. The striking male preponderance in patients with Morvan syndrome and CASPR2-Ab related disease is interesting. In previous study, CASPR2 mRNA has been found in the prostate tissue [11] and Morvan syndrome onset was observed after scrotal drainage in some cases [4]. These might indicated that the storage and release of relevant antibodies in the male productive system increase the titer of serum antibodies to cause Morvan syndrome [4, 11]. In the contrast, this case would add a valuable female resource to the mechanism study of Morvan syndrome and VGKC antibodies.

Although LGI1-Ab was commonly agreed with no association with neoplasm, no agreement on the relation between CASPR2-Ab and neoplasm has been reached. Previous studies have shown CASPR2-Ab positive patients were more likely to be found with neoplasm especially thymomas, accompanied with acetylcholine receptor antibodies and myasthenia gravis (MG) [4, 6, 11–13]. And CASPR2 has been reported to function as a tumor suppressor gene in glioma by Bralten et al. [14]. However, some other studies showed no neoplasm in a long follow-up in CASPR2-Ab positive patients [2, 9]. And there has been no evidence showing the difference of neoplasm concurrent rate between both CASPR2-Ab and LGI1-Ab positive patients and only CASPR2-Ab positive patients [4, 7]. With a short term follow-up, there has been no finding showed any neoplasm in this patient.

Most Morvan syndrome patients without neoplasm responded well to immunotherapy including IVIG, plasma exchange and corticosteroids, while patients who coexist with neoplasm mostly had a poor treatment outcome or prognosis [2, 4, 6]. In this case report, as for clinical syndromes, the patient got limited benefit from the initial IVIG treatment while got significant improvement with methylprednisolone pulse therapy, which was the same with other two cases [8, 15]. However, titres of CASPR2-Ab significantly reduced while LGI1-Ab turned negative with IVIG treatment, so the treatment effect of IVIG should not be overlooked either.

## Conclusions

In conclusion, this case provided a rare female case with typical Morvan syndrome as well as classical LE. It was showed from the detailed analysis of clinical course that the combination of LGI1-Ab and CASPR2-Ab might contribute to distinct phenotype of Morvan syndrome. With this case, we would like to draw your attention to the diverse and overlapping effects of these two autoantibodies. As to the treatment outcome and risk for neoplasm, we would have a continuous long follow-up on this patient.

## Consent

Written informed consent was obtained from the patient for publication of this case report and any accompanying images. A copy of the written consent is available for review by the Editor-in-Chief of this journal.

## Availability of data and materials

The datasets supporting the conclusions of this article are available in the zenodo repository.

Photos are available on https://zenodo.org/record/35473. And the video is available on https://zenodo.org/record/35474.

## Abbreviations

*PNH*: peripheral nerve hyperexcitability
*CASPR2*: contactin associated protein-like 2
*LGI1*: leucine-rich glioma inactivated protein 1
*LE*: limbic encephalitis
*n*: voltage-gated potassium channels
